# High-density genome-wide association study points out major candidate genes for resistance to infectious pancreatic necrosis in rainbow trout

**DOI:** 10.1186/s12711-025-00996-w

**Published:** 2025-09-25

**Authors:** Jonathan D’Ambrosio, Yoannah François, Thierry Morin, Sébastien Courant, Alexandre Desgranges, Pierrick Haffray, Bertrand Collet, Pierre Boudinot, Florence Phocas

**Affiliations:** 1 French Poultry and Aquaculture Breeders Association, SYSAAF, 35042 Rennes, France; 2 ANSES, Ploufragan-Plouzané-Niort Laboratory, VIMEP Unit, National Reference Laboratory (NRL) for Regulated Fish Diseases, FORTIOR Genetics Platform, National Research Infrastructure Emerg’In, 29280 Plouzané, France; 3 SARL Milin Nevez, 29610 Plouigneau, France; 4 Université Paris-Saclay, INRAE, UVSQ, VIM, 78350 Jouy-en-Josas, France; 5 Université Paris-Saclay, INRAE, AgroParisTech, GABI, 78350 Jouy-en-Josas, France

## Abstract

**Background:**

This study focuses on genetic resistance to infectious pancreatic necrosis (IPN), a highly contagious disease caused by an aquatic birnavirus (IPNV) which especially affects salmonids worldwide. The objectives were to estimate the heritability of IPN resistance and to fine map quantitative trait loci (QTL) using a Bayesian Sparse Linear Mixed Model to identify candidate genes possibly linked to IPN resistance in two successive generations from a French commercial strain of rainbow trout. For each generation, 2000 fish were experimentally exposed by bath to IPNV and mortalities were monitored daily during 5 weeks. All fish were genotyped using a medium-density 57 K single nucleotide polymorphism (SNP) chip and imputed to high-density genotypes (665 K SNPs).

**Results:**

The mean survival rate was 70% after 37 days, with a higher survival rate in the second generation compared to the first one (78% versus 61%). Heritability was moderate (~ 0.20). Approximately 74% of the genetic variance of IPN resistance was explained by several tens of SNPs. In total, 25 QTL were mapped on 10 chromosomes, of which 7 were detected with very strong evidence, on chromosomes 1, 14, 16 and 28. The most interesting QTL were associated to top SNPs with mean survival rate differences over 20% between the beneficial and detrimental homozygous genotypes. Those SNPs were all located within promising functional candidate genes on chromosome 1 (*uts2d*, *rc3h1*, *ga45b*) and chromosome 16 (*irf2bp*, *eif2ak2)*, which were all associated with regulation of inflammatory pathways. A key factor for the genetic differences in susceptibility to IPNV among fish is the dsRNA-dependent serine/threonine-protein kinase (PKR) encoded by the *eif2ak2* gene.

**Conclusions:**

All genes associated with the most significant QTL on chromosomes 1 and 16 are involved in the regulation of inflammatory pathways, strongly suggesting a central role of inflammation in IPN resistance in rainbow trout. These findings offer the possibility of marker-assisted selection for rapid dissemination of genetic improvement for IPN resistance.

**Supplementary Information:**

The online version contains supplementary material available at 10.1186/s12711-025-00996-w.

## Background

Infectious pancreatic necrosis (IPN) is a severe viral disease that affects salmonids worldwide. In rainbow trout farming, IPN was first described in 1940, in the USA [1], and reported in 1964 with catastrophic fry losses in French farms [2]. Its etiological agent, IPNV, is a double-stranded bi-segmented non-enveloped RNA birnavirus belonging to the Aquabirnavirus genus [3]. Its two genomic segments, called A and B, encode five viral polypeptides (VPs): VP1, an RNA-dependent RNA polymerase enzyme (segment B); VP2, the major capsid protein; VP3, an internal/minor capsid protein; VP4, a serine-lysine protease; and VP5, a non-structural protein (segment A). According to a phylogenetic classification based on VP2, seven genogroups have been defined to date [4]. IPNV infects over 63 different species of fish, molluscs, and crustaceans, and is highly prevalent in both farmed and wild fish [5]. In Europe, where a large number of countries cultivate salmonids, the majority of the farm isolates belong to genogroup 5 [4]. Mainly fry and juveniles less than six months old are affected by the disease, with up to 90% mortality on fry at start-feeding. The main symptoms are unusual behavior (abnormal swimming, i.e. spinning), skin melanism, abdominal distension, catarrhal lesions, and necrosis of the exocrine pancreas and liver tissues [5]. However, this highly contagious disease also affects farmed salmon at the post-smolt stage [3]. Survivors of an infection may become healthy adult carriers that can infect naïve fish [3]. The disease spreads horizontally via infected water and fish, but can also be transmitted vertically through eggs. Currently, no effective treatment is available. Several vaccine candidates (DNA, subunit, attenuated, inactivated, recombinant) have been developed and tested, and some commercial vaccines are available but it remains difficult to induce effective immune protection in the early stages that most affected by the virus [6–8]. However, good husbandry practices, such as maintaining high water quality and low stocking density, and avoiding mixing of batches, help to reduce disease incidence [3].

Selective breeding is an interesting alternative to control infectious diseases in salmonids [9]. Several studies have reported a significant genetic variation for resistance to IPN in Atlantic salmon, with moderate narrow-sense heritability estimates ranging from 0.31 to 0.45 [10]. In rainbow trout, estimates of heritability for IPN resistance are few, with values varying from 0.24 to 0.39 on the observed survival scale in a Chilean population [10, 11], depending on whether genomic or pedigree information was used to derive the estimates, and of 0.30 in a Norwegian breeding strain from AquaGen [12]. In Atlantic salmon, two research groups [13, 14] independently discovered a major QTL for IPN resistance on chromosome 26, using a few large full-sib fish groups from a Scottish and a Norwegian breeding population. The QTL turned out to be responsible for more than 80% of the genetic variation in IPN resistance both at the fry and the post-smolt life stages in Atlantic salmon [15, 16]. This QTL for IPN resistance was an ideal case for marker-assisted selection (MAS) programs in Atlantic salmon, as it explained a large fraction of the phenotypic variance of a trait with high economic value, and was still segregating with an intermediate allele frequency in many populations, with the high-resistance allele being partly dominant over the low-resistance allele [17]. In Norway, MAS for IPN resistance has contributed to a 75% decline in the number of IPN-outbreaks within a few years from 2009 to 2013 [18]. The functional mutation underlying this QTL was identified in the *epithelial cadherin* gene (*cdh1*). In a co-immunoprecipitation assay, CDH1 was found to bind to IPNV virions, indicating that the protein was involved in internalization of the virus [16]. To test this hypothesis, *cdh1* KO SHK-1 cells were generated by [19], who demonstrated that *cdh1* was not essential for the entry of IPNV into salmon cells, nor for successful IPNV replication. In contrast, both knockout and chemical inhibition of the *NEDD-8 activating enzyme E1* (*nae1*) gene resulted in significant reduction in IPNV replication in cell lines, suggesting that *nae1* was the causative gene underlying the major QTL for resistance to IPNV in salmon [19]. However, a new variant of IPNV was recently detected in Norway, with mutations that have made the virus capable to cause disease even in the genetically IPN resistant fish for the major QTL [20].

In rainbow trout, the few QTL identified in the literature were not located in the vicinity of the major QTL for IPN resistance identified in salmon. The use of low-resolution molecular markers has shown the presence of two genomic regions associated with resistance and susceptibility to IPN in rainbow trout [21, 22]. In two families, one hybrid family between an IPN resistant and an IPN susceptible Japanese rainbow strains, and a backcross family, two QTL were found on linkage groups LG 3 and LG 22 [21, 22], corresponding to chromosomes 14 and 16, respectively [23]. These two QTL are on linkage groups that have no clear homology with the linkage groups in which QTL were identified for IPN resistance in Atlantic salmon [24, 25]. The first medium-density GWAS, using SNPs on a small set of 721 phenotyped and genotyped Chilean rainbow trout from 58 full-sib families [11], provided some moderate evidence for QTL, mainly on chromosomes 5, 13, 21, and 23. In addition, based on a batch-challenge test and 57 K SNP genotyping of 1723 rainbow trout from 46 full-sib families, a US patent was deposited by AquaGen AS [26], revealing a significant IPN QTL region in chromosome 1, with the 3 most significant SNPs being located between 16 and 19 Mb on chromosome 1 of the Arlee reference genome [27].

As some French rainbow trout farms encountered major issues with IPN outbreaks in the 2010s, the Milin Nevez breeding company was willing to select its commercial strain for resistance to IPNV. For this purpose, IPNV challenge tests were conducted in 2018 and 2020 on two cohorts of collaterals of the selection candidates and fish were genotyped with the 57 K to run genomic selection. Some parents and sibs of the challenged fish were genotyped with a high-density SNP chip that is now available for rainbow trout [28]. The objectives of this research were to estimate the heritability of IPN resistance and to perform QTL fine mapping to identify candidate genes possibly linked to IPN resistance in two successive generations from a French commercial strain of rainbow trout.

## Methods

### Animals

The fish challenged belonged to the 8th (G8) and 9th (G9) generations of a commercial breeding program developed by the Milin Nevez breeding company (Plouigneau, France), as they were produced by breeders from the 7th and the 8th generations of selection, respectively. Therefore, the breeders from the 8th generation of selection (producing the G9 fish) were sibs of the challenged fish in G8. For G8, the experimental stock was established from 81 dams and 91 sex-reversed neomales with 10 independent full-factorial mating designs (~ 8 dams × 8–10 neomales in each factorial). For G9, the experimental stock was established from 90 dams and 98 neomales with 10 independent full-factorial mating designs (9 dams × 8–10 neomales in each factorial).

A batch of eyed eggs was transferred to the SYSAAF-ANSES FORTIOR Genetics platform (Plouzané, France) at about 200–250 degree-days. Fry were reared in tanks in an opened flow-through system with filtered freshwater at 10 °C ± 2 temperature. About 15 to 20 days after start feeding (about 700 degree-days, or 1.5 g), fry were transferred to adapted tanks for the infectious challenges. Fish were individually identified post-challenge using the DNA barcode of the individual fin sampling.

### Challenge tests

Fish were challenged in year 2018 for G8 and in 2020 for G9, respectively. Both infectious challenges were conducted the same way. Each generation, 2000 fry were exposed to a strain of IPNV isolated from an affected farm during a bath in static aerated freshwater at 10 °C ± 2 containing 1.10^5^ Tissue Culture Infectious Dose (TCID)_50_/ml of virus. For G8 and G9, the strains used were identified as NN193 (isolated in 2015) and NPI11125 (isolated in 2019), respectively, both belonging to genogroup 5. After 3 h, water was restarted in open circuit, with a minimum hourly renewal, at 10 °C ± 2. The two challenges lasted 37 and 36 days post-infection (dpi), respectively, during which fish were monitored daily and fed at least twice a day. Days of mortality were recorded and DNA samples were individually collected for all dead fish (caudal fin sampled and stored in alcohol tubes in 4 °C). At the end of the challenges, survivors were sacrificed using a lethal dose of Eugenol (180 ppm; Fili@Vet Réseau Cristal) and their DNA samples were also collected. To control the sanitary status of dead (infected) and control (uninfected) fish at the mortality peak, broad virological (NRL for regulated fish diseases) and bacteriological (Labocea, Quimper) analyses were performed on 10 samples in each group. It allows to check the absence of co-infection as well the absence of IPNV in control fish and its presence in dead ones. IPNV detection was carried out according to the AFNOR UN 47–222 standard by culture on EPC (epithelioma papulosum cyprinid), BF2 (Bluegill fry) or CHSE (Chinook salmon embryo) cell lines, followed by seroneutralization.

### Genotyping and imputation

Fin samples from 1878 (G8) and 1992 (G9) challenged fish and their 372 parents were sent to the INRAE genotyping platform Gentyane (Clermont-Ferrand, France) for DNA extraction and genotyping. The 3870 challenged fish, as well as the 186 parents of G9 were genotyped for 57,501 SNPs using the medium-density (MD) Rainbow Trout Axiom^®^ 57 K SNP array.

The 186 parents of G8, as well as 95 sibs (dams of the 10th generation of selection, produced by 100 G8 parents) of the G9 challenged fish, were genotyped for 664,531 SNPs using the high-density (HD) Rainbow Trout Axiom^®^ 665 K SNP array [28]. Then, SNPs with probe polymorphism and multiple locations on the Arlee reference genome assembly (GCA_013265735.3; [27]) were discarded, as described in [28].

For both MD and HD genotypes, PLINK v1.9 software [29, 30] was used to keep only SNPs with deviation from Hardy–Weinberg equilibrium with a p-value > 0.000001, a minor allele frequency greater or equal to 5%, and both SNP and sample call rates above 98%. After quality control, 409,786 and 27,130 SNPs were retained for the HD and MD genotypes, respectively. In total, respectively 162 and 2 fish samples did not pass quality control for the MD and HD genotypes.

Parentage assignment was done using 1000 randomly sampled SNPs, using the R package APIS [31, 32] with the positive assignment error rate set to 1%. The successful assignment rate was 100% for G9 offspring and 92.6% for G8 offspring. Fish not assigned were kept for imputation with unknown pedigree.

Imputation of the MD genotypes into HD genotypes for the 3707 offspring (1757 G8, 1950 G9) was conducted using the FIMPUTE3 software [33], utilizing quality-filtered genotypes and pedigree information from parents. Correctness of imputation was checked by mendelian error testing. On average, there were 6 mendelian errors per SNP, with a maximum of 3 errors (< 1% of the progeny) observed for 75% of the 409,786 SNPs. A last quality filter was used to remove SNPs with over 100 mendelian errors after imputation. In total 394,101 SNPs were retained for the analysis of HD genotypes on 3707 phenotyped progeny, with 3 mendelian observed on average per SNP and a maximum of 2 errors for 75% of the SNPs.

### Estimation of genetic parameters

Variance components for IPN resistance were estimated using the restricted maximum likelihood method applied to a (G)BLUP linear animal model and the AIREML algorithm in BLUPF90 software [34]. The generation effect was the only fixed effect included in the model. In total, 44,367 animals were related through the pedigree relationship matrix for BLUP evaluation, tracing back 9 generations of ancestors of the 3855 phenotyped animals. For GBLUP evaluation, the pedigree relationship matrix was replaced by a realized relationship matrix combining pedigree and genomic information [35].

### QTL mapping

The genome-wide association study (GWAS) was based on a Bayesian Sparse Linear Mixed Model (BSLMM) that assumes that all SNPs have at least a relatively small effect but also that some SNPs may have a large effect [36]. The BSLMM was applied to the IPN challenge resistance phenotypes (here, a binary trait corresponding to the status ‘dead’ or ‘alive’), corrected by the generation effects estimated under the linear BLUP model previously described. The resulting residuals were treated as the vector of phenotypes **y** described as follows:

1 $$ {\mathbf{y}} = {\mathbf{1}}_{{\mathbf{n}}} \mu + {\mathbf{X\beta }} + {\mathbf{u}} + {\upvarepsilon } $$

where **1**_**n**_ is an n-vector of 1 s, µ is a scalar representing the mean, **X** is an n × p matrix of genotypes (covariates coded as 0, 1 or 2 depending on the number of copies of the reference allele at each marker) measured on n individuals at p = 394,101 SNPs, **β** is the corresponding p-vector of the SNP effects; **u** is a vector of random additive genetic effects distributed according to *N*(**0**, **K**σ^2^_b_), with σ^2^_b_ the additive genetic variance and **K** the genomic relationship matrix derived using the p SNPs; and **ε** is a n-vector of residuals* N*(**0**, **I** σ^2^_e_), where σ^2^_e_ is the variance of the residual errors.

Assuming **K** = **XX**^**T**^ /p, the SNP effect sizes can be decomposed into two parts: α that captures the small effects that all SNPs have, and β that captures the additional effects of some large effect SNPs. In this case, u = Xα can be viewed as the combined effect of all small effects, and the total effect size for a given SNP is γ_i_ = α_i_ + β_i_. The individual SNP effects γ_i_ are sampled from a mixture of two normal distributions, γ_I_ ∼ π N(0, σ^2^_a_ + σ^2^_b_) + (1 − π) N(0,σ^2^_b_), where σ^2^_b_ is the variance of small additive genetic effects, σ^2^_a_ is the additional variance associated to large effects and π is the proportion of SNPs with large effects.

The BSLMM was implemented using the Genome-Wide Efficient Mixed Model Association (GEMMA) software based on a Markov chain Monte Carlo (MCMC) method applied to a linear mixed model (‘-bslmm 1’ option) as proposed by [36] for a binary survival trait. Treating a binary trait as a continuous quantitative trait is justified by recognizing the linear model as a first order Taylor approximation of a generalized linear model, as well as by the robustness of the linear model to model misspecification [36].

A total of 2.2 million iterations (-s option) were performed with a burn-in of 200,000 cycles and results were saved every 100 iterations for further analysis. In addition, to ensure convergence of the distribution of the hyper-parameter π, the minimum and maximum numbers of SNPs that was sampled to be included in the model were set to 1 (-smin option) and 300 (-smax option), respectively. We ran 3 chains of the MCMC with 3 different initial random seeds in order to check convergence of the estimates.

The MCMC sampling of all parameters values from the posterior distribution allows all SNP effects $\widetilde{\beta }$ to be estimated, but also the hyper-parameter π and the posterior inclusion probability (PIP) of each SNP, which quantifies the proportion of samples in which the SNP has a large effect in the model. This PIP value indicates the strength of the evidence that the SNP has to be included in the model and can therefore be used for QTL mapping.

To define a minimum threshold for the strength of the evidence for a given SNP, Stephens and Balding [37] proposed to calculate the Bayesian Factor BF = [PIP/(1-PIP)] / [ π/(1- π)]. The logBF was computed as twice the natural logarithm of the BF to be in the usual range of likelihood ratio test values. A minimum threshold value of logBF = 10 (BF ≈ 150) was used for defining a top SNP indicating strong evidence for a QTL [38]. A more stringent value of logBF = 12 (i.e. BF ≈ 400) was considered for pointing out very strong evidence for a QTL [39].

The GWAS results were visualized via a Manhattan plot, with negative values for logBF were set to 0. To account for differences in allele frequencies and linkage disequilibrium between SNPs, credibility intervals were determined by including any SNP in a QTL region as soon as it had a logBF ≥ 7 within a 100 kb sliding window from the top SNP with evidence for the QTL (i.e. logBF ≥ 10).

Genes within QTL regions were annotated using the NCBI *O. mykiss* Arlee genome assembly USDA_OmykA_1.1. (GCA_013265735.3, [27]). In addition to NCBI gene summaries, functional information for genes was extracted from the human gene database GeneCards® (https://www.genecards.org/), which also includes protein summaries from UniProtKB/Swiss-Prot (https://www.uniprot.org/uniprotkb/).

### Effects of QTL genotypes

To assess how the QTL genotypes affect the survival rate, the estimates of the effects of top SNPs in each QTL region were calculated. To do so, we adjusted the observed survival rates for generation effects in order to be able to compare the two challenge test results. Then, we averaged these residuals and added the mean survival rate derived across the two generations to facilitate the discussion of the results. We analyzed these estimates for each QTL as well as for combinations of 2 QTL and 3 QTL. For each top SNP, we checked that these raw estimates were very close to BLUP estimates derived under a BLUP Animal model accounting for both the fixed effects of the generation and of the genotypes at the top SNP.

## Results

### Challenge tests

**Fig. 1:**
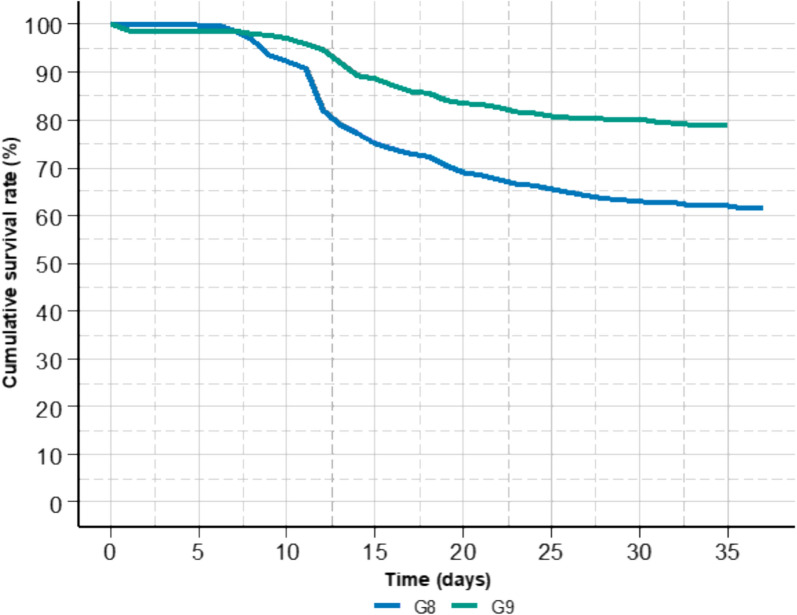
Evolution of the Kaplan–Meier cumulative survival rate during the challenge tests of the two cohorts

The mean survival rate was 70% after 37 days, with a higher survival rate in G9 compared to G8 (78% versus 61%). Mortality kinetics were relatively similar between the two cohorts, but with a more rapid decline in survival between 8 and 15 days for G8 (Fig. 1).

The presence of IPNV was confirmed in dead fry by observation of cytopathic effects in cell cultures and identification by seroneutralization. Bacteriological analyses did not reveal any pathogenic germs. No infectious agent was detected in uninfected control animals.

### Genetic architecture of IPN resistance

Heritability estimates of survival were very moderate, close to 0.20 on the observed scale for both the BLUP and GBLUP models (Table 1). The corresponding parameter (PVE) under BSLMM was estimated at a slightly lower value that was consistent across the 3 MCMC runs. Across the 3 MCMC runs of BSLMM, approximately 74% of the total genetic variance of IPN resistance was explained by on average 63 SNPs fitted with a large effect in the model.

**Fig. 2:**
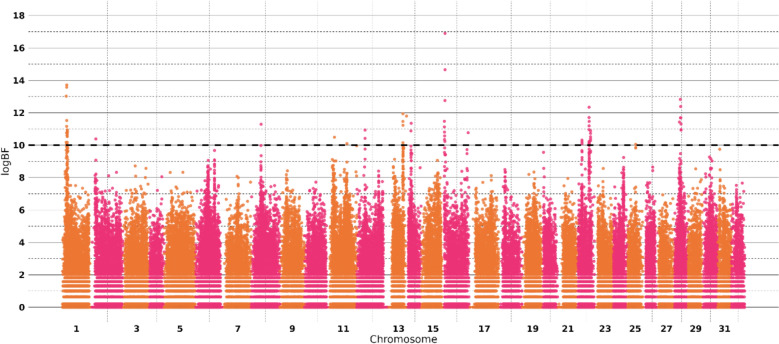
Manhattan plot showing associations between SNPs and IPN resistance. The dashed line represents a threshold value of logBF = 10, indicating a strong association of the SNP with IPN resistance.

In the GWAS, 25 QTL were identified in regions on chromosomes 1, 2, 8, 11, 12, 13, 14, 16, 22, and 28 (Fig. 2). They included 53 SNPs with a strong evidence (logBF > 10), meaning a Bayes Factor BF > 150 across at least 2 MCMC runs (Table 2). Most of these SNPs were located within NCBI annotated genes (Table 2).

**Table 1:** Heritability estimates for IPN resistance on the observed scale according to the genetic model

Model – heritability symbol	PBLUP – h^2^	GBLUP – h^2^	BSLMM—PVE
Mean estimate (± SE)	0.206 (± 0.005)	0.190 (± 0.023)	0.154 (± 0.018)

Major evidence (logBF ranging from 14.7 to 16.9 for two SNPs) was found on chromosome 16 for a QTL (Q16.3) harbouring the region of *eukaryotic translation initiation factor 2-alpha kinase 2* (*eif2ak2*) gene. This gene, also known as *pkr (protein kinase* *RNA-activated*), is a conserved interferon stimulated gene that mediates virus induced protein shut off via inhibition of translation, promotes apoptosis, fosters type I IFN responses induced by some viruses, and modulates inflammation through activation of MAPK and NFkB pathways [40]. At the two top SNPs located within *pkr* (Table 2), the major allele, G for both SNPs, corresponded to the resistant allele (R) and the minor allele A was the susceptible allele (S) with a MAF of 15.9%. Of note, the major allele G for the first top SNP, Affx-1237475214, is the Arlee alternative allele, while the major allele G for the second top SNP, Affx-1248466717, is the Arlee reference allele (see Additional file 1: Table S1).

**Table 2:** Top SNPs, Bayes Factor statistics (logBF), credibility intervals (CI), and candidate genes for IPN resistance in rainbow trout

Chr	ID SNP probe	Position (Mb)	MAF	logBF_1	logBF_2	logBF_3	QTL ID	CI start (Mb)	CI end (Mb)	GENE NAME (at top SNP)
1	AX-564714180	11.816099	0.331	14.2	11.1	13.0	Q1.1	11.801	11.884	leucine-rich repeat-containing protein 15 (lrr5)
1	AX-578239392	11.833088	0.116	10.1	< 10	10.2	Q1.1			urotensin 2 domain containing (uts2d)
1	AX-564726411	12.875479	0.467	10.4	10.1	10.8	Q1.2	12.858	12.875	choline transporter-like protein 5-B (SLC44a5B)
1	AX-89957079	13.108828	0.486	10.8	10.2	10.0	Q1.3	12.980	13.109	MAP kinase-interacting serine/threonine-protein kinase 1 (mknk1)
1	AX-89918646	13.330100	0.490	< 10	11.5	11.5	Q1.4	13.225	13.425	growth hormone-releasing hormone receptor-like
1	AX-564768903	13.356636	0.474	12.5	12.9	13.6	Q1.4			FYVE and coiled-coil domain-containing protein 1 (fycol1)
1	AX-564733791	13.424509	0.474	12.7	13.1	13.7	Q1.4			T-cell acute lymphocytic leukemia protein 1 homolog
1	AX-564559120	14.223187	0.262	< 10	10.3	10.6	Q1.5	14.220	14.249	axonemal dynein light chain domain-containing protein 1
1	AX-564690966	15.113131	0.132	< 10	10.2	10.9	Q1.6	15.070	15.513	close to uncharacterized lncRNA
1	AX-578240074	15.125177	0.132	< 10	10.6	10.9	Q1.6			no gene
1	AX-578242608	15.150954	0.132	10.0	< 10	10.8	Q1.6			ring finger and CCCH-Type domains 1 (rc3h1)
1	AX-578242826	15.491303	0.132	10.2	< 10	10.2	Q1.6			no gene
1	AX-564725443	15.857950	0.138	< 10	10.6	10.8	Q1.7	15.762	15.858	growth arrest and DNA-damage-inducible protein GADD45 beta (ga45b)
2	AX-564898476	17.152576	0.243	10.2	< 10	10.4	Q2.1			double-stranded RNA-specific adenosine deaminase-like
8	AX-298393569	31.660427	0.414	10.0	10.9	11.3	Q8.1	31.617	31.706	uncharacterized protein C14orf132
11	AX-564602468	14.522132	0.290	10.5	10.3	10.5	Q11.1	14.522	14.657	brefeldin A-inhibited guanine nucleotide-exchange protein 1 (arfgef1)
12	AX-564618193	26.056954	0.451	10.5	10.2	10.4	Q12.1	26.047	26.057	ring finger protein 128a
13	AX-578666916	48.670239	0.395	10.0	10.4	11.5	Q13.1	48.670	49.070	nucleobindin-2
13	AX-564888254	48.724724	0.401	10.9	10.7	11.9	Q13.1			tripartite motif-containing protein 16-like (TRIM16L pseudogene)
13	AX-579021194	48.770297	0.395	10.0	< 10	10.2	Q13.1			myosin-binding protein C, fast-type
13	AX-578667058	49.069672	0.408	10.3	10.3	11.2	Q13.1			uncharacterized LOC110486659
13	AX-564888311	51.394091	0.328	10.9	< 10	10.9	Q13.2	none	none	prostaglandin E2 receptor EP1 subtype-like
13	AX-578669743	60.516081	0.236	12.3	10.9	11.8	Q13.3	none	none	ras-related protein Rab-26 (rab26)
14	AX-298114291	9.993684	0.497	12.3	10.2	12.3	Q14.1	9.727	10.000	fibroblast growth factor 13 (fgf13)
16	AX-565279753	0.025948	0.223	11.1	11.7	11.5	Q16.1	0.026	0.863	myosin heavy chain, fast skeletal muscle
16	AX-564709071	0.050475	0.226	10.0	10.1	11.1	Q16.1			myosin heavy chain, fast skeletal muscle
16	AX-564710565	0.402703	0.241	11.1	10.1	10.8	Q16.1			uncharacterized lncRNA LOC110491220
16	AX-564711785	0.773151	0.242	10.2	10.7	10.6	Q16.1			signal-induced proliferation-associated 1-like protein 2
16	AX-171601028	1.982167	0.265	13.9	12.3	12.8	Q16.2	none	none	mRNA-interferon regulatory factor 2-binding protein 2-A (irf2bp2)
16	AX-564712030	2.258205	0.159	15.5	14.7	16.9	Q16.3	2.258	2.289	eukaryotic translation initiation factor 2-alpha kinase 2 (eif2ak2 / pkr)
16	AX-578720839	2.287808	0.159	15.6	14.8	14.7	Q16.3			eukaryotic translation initiation factor 2-alpha kinase 2 (eif2ak2 / pkr)
22	AX-564933197	13.155734	0.202	10.4	< 10	10.2	Q22.1	13.153	13.246	ephrin-B2a (efnb2a)
22	AX-578884865	35.427230	0.187	10.6	< 10	11.0	Q22.2	35.396	36.070	EGF-like domain-containing protein
22	AX-565407488	35.484747	0.186	10.9	< 10	11.7	Q22.2			no gene
22	AX-578882935	35.490275	0.187	11.3	10.3	10.1	Q22.2			no gene
22	AX-565406239	35.572115	0.186	11.3	< 10	11.2	Q22.2			no gene
22	AX-578882969	35.577677	0.185	11.5	11.2	12.3	Q22.2			no gene (next: ras-related protein Ral-B: 35,697,586..35,738,804)
22	AX-89945075	35.699314	0.254	10.0	< 10	10.2	Q22.2			inhibin beta B chain (LOC110501797)
22	AX-565406294	35.839289	0.246	10.2	10.8	11.5	Q22.2			no gene
22	AX-564927499	35.909034	0.207	10.8	< 10	10.0	Q22.2			no gene (next: zinc finger protein GLI2: 35,943,510..36,060,137)
22	AX-565133771	40.326787	0.422	11.0	11.0	10.5	Q22.3	40.327	40.402	no gene
22	AX-565408929	40.327296	0.423	10.3	< 10	10.3	Q22.3			no gene
22	AX-565407271	40.343344	0.422	10.7	< 10	10.7	Q22.3			no gene
22	AX-565407288	40.394178	0.422	10.0	< 10	10.4	Q22.3			antifreeze protein Maxi-like (LOC118943589)
22	AX-565407289	40.394713	0.422	11.0	11.0	10.9	Q22.3			R3H domain-containing protein 1
28	AX-89963799	15.342242	0.372	11.0	< 10	11.4	Q28.1	15.342	15.342	no gene (close: forkhead box protein Q1:15,339,285..15,341,799)
28	AX-578991831	18.009225	0.381	13.4	11.8	12.8	Q28.2	17.990	18.413	rho GTPase-activating protein 21 (arhgap21)
28	AX-565484548	18.413047	0.375	11.8	11.4	11.7	Q28.2			myosin-6
28	AX-565011274	19.207695	0.169	12.4	11.8	12.4	Q28.3	19.207	19.730	probable G-protein coupled receptor 141 (gpr141)
28	AX-565009231	19.729884	0.238	11.2	< 10	11.7	Q28.3			sickle tail protein homolog (LOC110508698)
28	AX-565484597	20.472799	0.104	10.1	10.6	10.9	Q28.4	20.473	20.586	E3 ubiquitin-protein ligase RNF152-like (rnf152)
28	AX-298518121	20.494901	0.104	11.3	< 10	11.0	Q28.4			uncharacterized LOC110508708
28	AX-565009263	20.585570	0.152	12.0	11.6	11.3	Q28.4			XK-related protein 4 (xkr4)

Six other QTL with very strong evidence (logBF > 12 for at least 2 out of the 3 MCMC runs) were detected on chromosomes 1 (Q1.1, Q1.4), 14 (Q14.1), 16 (Q16.2), and 28 (Q28.2, Q28.3). Some top SNPs were also identified within genes for all six QTL (Table 2).

Several genes associated with the identified QTL are orthologous to genes that control either the type I IFN response and/or inflammation (*uts2d* and *rc3h1* on chr 1; *rab26* on chr 13; *irf2bp2* located in Q16.2 close to *pkr* on chr 16; *gpr141* and *rnf52*, respectively in Q28.3 and Q28.4 on chr 28). Located in Q1.1, *uts2d* is a homolog of the mammalian *urotensin-2* that induces a number of pro-inflammatory genes, including TNF-α, IL-1β, IFN-γ, IL-8, and leukotriene C4, or activates pathways like NFκB and IRF3 signaling [41]. In Q1.6, the gene *rc3h1* (also named *roquin-1*) encodes for an anti-inflammatory and regulatory factor [42, 43].

The other genes associated with the identified QTL played various roles in the biology of the cell thatmight be connected to the virus cycle or to antiviral processes: choline/phospholipid metabolism (s*lc44a5b* in Q1.2; *xkr4* in Q28.4), translation control (*mknk1* in Q1.3), intracellular vesicular trafficking (*fyco1* in Q1.4; *argef1* in Q11.1), and actine remodelling (*arhgap21* in Q28.2 on chr 28).

### QTL effects on IPN resistance

Survival rates were calculated from all 53 top SNPs in each of the two generations. Average differences in survival rate between the two homozygous genotypes as well as between the major homozygote and the heterozygote are presented in Table 3. The phenotypic estimates of these differences were in close agreement with corresponding BLUP estimates (r = 0.98) with deviations between the two estimates below 3 points of survival between the two homozygous genotypes, except for top SNPs identified in Q1.6 and Q1.7. For these SNPs, really stronger differences in survival between the two homozygotes were estimated by BLUP (~ 36%) compared to the phenotypic estimates (~ 30%, Table 3).

**Table 3:** Survival rates differences between genotypes at the top SNPs for resistance to IPNV, along with their frequencies

Chr	ID SNP probe	position (Mb)	Major allele	Minor allele	QTL ID	Frequency of major allele homozygotes	Frequency of minor allele homozygotes	Difference in % survival between homozygotes for the major and minor alleles		Difference in % survival between the major allele homozygotes and heterozygotes	
								Phenotypic estimate	BLUP estimate	Phenotypic estimate	BLUP estimate
1	AX-564714180	11.816099	C	T	Q1.1	0.34	0.18	13.9	15.3	3.7	4.1
1	AX-578239392	11.833088	G	A	Q1.1	0.74	0.02	25.5	28.1	8.7	9.5
1	AX-564726411	12.875479	C	A	Q1.2	0.42	0.13	− 12.0	− 13.3	− 14.3	− 14.9
1	AX-89957079	13.108828	A	G	Q1.3	0.16	0.35	13.7	15.4	0.1	1.0
1	AX-89918646	13.330100	T	G	Q1.4	0.16	0.36	13.7	15.3	0.1	0.8
1	AX-564768903	13.356636	A	C	Q1.4	0.18	0.33	15.5	16.6	2.6	3.3
1	AX-564733791	13.424509	G	T	Q1.4	0.18	0.33	15.7	16.7	2.5	3.2
1	AX-564559120	14.223187	C	T	Q1.5	0.50	0.09	17.3	19.4	5.0	5.4
1	AX-564690966	15.113131	G	A	Q1.6	0.72	0.02	29.8	36.4	6.8	8.4
1	AX-578240074	15.125177	C	A	Q1.6	0.72	0.02	29.8	36.4	6.8	8.4
1	AX-578242608	15.150954	G	T	Q1.6	0.72	0.02	29.8	36.4	6.9	8.4
1	AX-578242826	15.491303	C	A	Q1.6	0.72	0.02	27.4	33.6	7.4	8.8
1	AX-564725443	15.857950	G	A	Q1.7	0.71	0.02	30.0	36.3	6.5	8.1
2	AX-564898476	17.152576	C	T	Q2.1	0.55	0.06	11.4	11.0	4.4	4.3
8	AX-298393569	31.660427	A	C	Q8.1	0.37	0.15	− 8.9	− 8.9	− 5.9	− 6.3
11	AX-564602468	14.522132	G	A	Q11.1	0.56	0.06	− 13.0	− 10.6	− 6.0	− 4.8
12	AX-564618193	26.056954	C	T	Q12.1	0.30	0.19	7.3	7.7	5.2	5.8
13	AX-578666916	48.670239	A	G	Q13.1	0.32	0.17	8.5	11.1	6.7	7.3
13	AX-564888254	48.724724	T	C	Q13.1	0.31	0.17	8.3	10.8	6.8	7.3
13	AX-579021194	48.770297	C	A	Q13.1	0.32	0.17	8.3	10.8	6.8	7.3
13	AX-578667058	49.069672	G	A	Q13.1	0.30	0.19	8.6	11.0	6.0	6.7
13	AX-564888311	51.394091	G	T	Q13.2	0.38	0.13	8.5	10.0	7.6	7.8
13	AX-578669743	60.516081	T	G	Q13.3	0.50	0.07	6.8	11.4	4.4	6.6
14	AX-298114291	9.993684	C	A	Q14.1	0.24	0.26	12.3	11.8	6.2	6.2
16	AX-565279753	0.025948	A	C	Q16.1	0.54	0.07	19.2	19.0	10.5	9.5
16	AX-564709071	0.050475	G	T	Q16.1	0.54	0.07	19.0	18.6	10.4	9.4
16	AX-564710565	0.402703	T	C	Q16.1	0.49	0.08	18.5	17.4	9.3	8.0
16	AX-564711785	0.773151	G	A	Q16.1	0.49	0.08	18.5	17.4	9.4	8.1
16	AX-171601028	1.982167	T	C	Q16.2	0.49	0.10	19.6	16.6	10.6	8.5
16	AX-564712030	2.258205	G	A	Q16.3	0.64	0.04	24.1	22.4	12.4	10.2
16	AX-578720839	2.287808	G	A	Q16.3	0.64	0.04	24.1	22.4	12.4	10.2
22	AX-564933197	13.155734	C	T	Q22.1	0.63	0.04	− 9.7	− 13.1	− 6.4	− 5.4
22	AX-578884865	35.427230	C	T	Q22.2	0.64	0.03	12.3	10.3	7.9	7.5
22	AX-565407488	35.484747	A	G	Q22.2	0.64	0.03	12.0	9.7	8.1	7.6
22	AX-578882935	35.490275	T	C	Q22.2	0.64	0.03	11.9	9.6	8.0	7.5
22	AX-565406239	35.572115	A	G	Q22.2	0.64	0.03	11.5	9.3	8.1	7.6
22	AX-578882969	35.577677	G	A	Q22.2	0.64	0.03	12.1	9.8	8.1	7.7
22	AX-89945075	35.699314	G	A	Q22.2	0.55	0.07	11.2	11.4	6.2	6.5
22	AX-565406294	35.839289	A	C	Q22.2	0.55	0.06	12.5	13.0	6.0	6.2
22	AX-564927499	35.909034	T	C	Q22.2	0.60	0.05	13.8	14.3	5.8	5.8
22	AX-565133771	40.326787	A	C	Q22.3	0.31	0.19	10.6	12.5	3.0	5.1
22	AX-565408929	40.327296	C	A	Q22.3	0.31	0.19	10.4	12.2	3.0	5.1
22	AX-565407271	40.343344	A	G	Q22.3	0.31	0.20	10.4	12.3	2.5	4.7
22	AX-565407288	40.394178	A	G	Q22.3	0.31	0.19	10.5	12.3	3.0	5.1
22	AX-565407289	40.394713	A	G	Q22.3	0.31	0.20	10.6	12.4	2.9	5.1
28	AX-89963799	15.342242	T	G	Q28.1	0.38	0.14	− 10.2	− 10.7	− 4.7	− 4.1
28	AX-578991831	18.009225	G	T	Q28.2	0.40	0.15	13.2	11.0	4.5	4.2
28	AX-565484548	18.413047	T	C	Q28.2	0.41	0.14	13.2	11.4	3.9	4.0
28	AX-565011274	19.207695	T	C	Q28.3	0.69	0.03	− 11.7	− 12.6	− 6.7	− 7.5
28	AX-565009231	19.729884	A	G	Q28.3	0.59	0.05	− 9.3	− 12.7	− 4.1	− 5.5
28	AX-565484597	20.472799	A	C	Q28.4	0.81	0.01	− 8.2	− 9.5	− 8.6	− 9.3
28	AX-298518121	20.494901	T	G	Q28.4	0.81	0.01	− 8.2	− 9.5	− 8.6	− 9.3
28	AX-565009263	20.585570	A	G	Q28.4	0.70	0.02	− 12.6	− 15.2	− 5.9	− 7.0

The SNPs identified as having the strongest phenotypic effect (> 24% between the two homozygotes, Table 3) were located in 3 QTL on chr 1 (Q1.1, Q1.6, Q1.7) and in Q16.3 on chromosome 16. All the 13 SNPs identified in the 7 QTL detected on chromosome 1 exhibited at least a significant difference of 12% in survival rates between the two homozygous genotypes. These 13 SNPs also showed complete or incomplete beneficial dominance effect of the major alleles, except for Q1.2, for which a complete beneficial dominance effect was observed for the minor allele of AX-564726411 (Table 3).

The two largest differences (29.8 and 30.0%) in the survival rates of homozygous genotypes were observed for the SNPs AX-578242608 and AX-564725443, respectively located in the *rc3h1* gene (Q1.6) and close to *gadd45b* (*growth arrest and DNA-damage-inducible protein GADD45 beta)* gene (Q1.7). For these two SNPs, survival rates for individuals with favorable homozygous genotypes were 64 and 81% for, respectively, G8 and G9, while the rates dropped to 29 and 56% for the unfavorable homozygous genotypes (see Additional file 1: Table S2). The top SNPs across these two QTL are in very strong linkage disequilibrium (LD = 0.946) (see Additional file 1: Table S3).

Regarding the 3 QTL on chromosome 16, all the 7 top SNPs exhibited at least a difference of 18.5% in survival rates between the two homozygous genotypes (Table 3) and showed pure additive genetic effects, as the survival rate of the heterozygotes was very close to the mean survival rate of the two homozygotes for each of these 7 SNPs. Fish with resistant homozygous genotypes for the main QTL (Q16.3) on chromosome 16 had a survival rate of 67% in G8 and 83% in G9, compared to 38% in G8 and 63% in G9 for individuals with the susceptible homozygous genotypes (see Additional file 1: Table S2).

For all top SNPs in the 15 remaining QTL regions (on chromosomes 2, 8, 11, 13, 14, 22 and 28), the differences in survival rates between homozygote genotypes were moderate, with absolute values ranging from 7 to 13%. The favorable genotypes were the major homozygous ones, except for the SNPs corresponding to Q8.1, Q11.1, Q22.1, Q28.1, Q28.3, and Q28.4, for which the beneficial alleles were the minor ones (see Table 3).

The very best 2-QTL combination of resistant genotypes exhibited mean survival rates over 90% (see Additional file 1: Table S4), but were rare in the population (< 2%), weakening the confidence in these estimates. These combinations associated Q1.4 with Q1.3 or Q1.5, and Q8.1 with Q28.1. The combination of favorable genotypes for QTL on chromosomes 16 and 1 showed survival rates ranging from 77.2 to 84.0%. Among these latter combinations, the highest survival rate was observed for favorable genotypes associated with Q1.2 and Q16.2; the beneficial combination between Q1.6 and Q16.3 was more frequent (45% *versus* 24%) and exhibited a mean survival rate of 77.6% (see Additional file 1: Table S4).

**Fig. 3:**
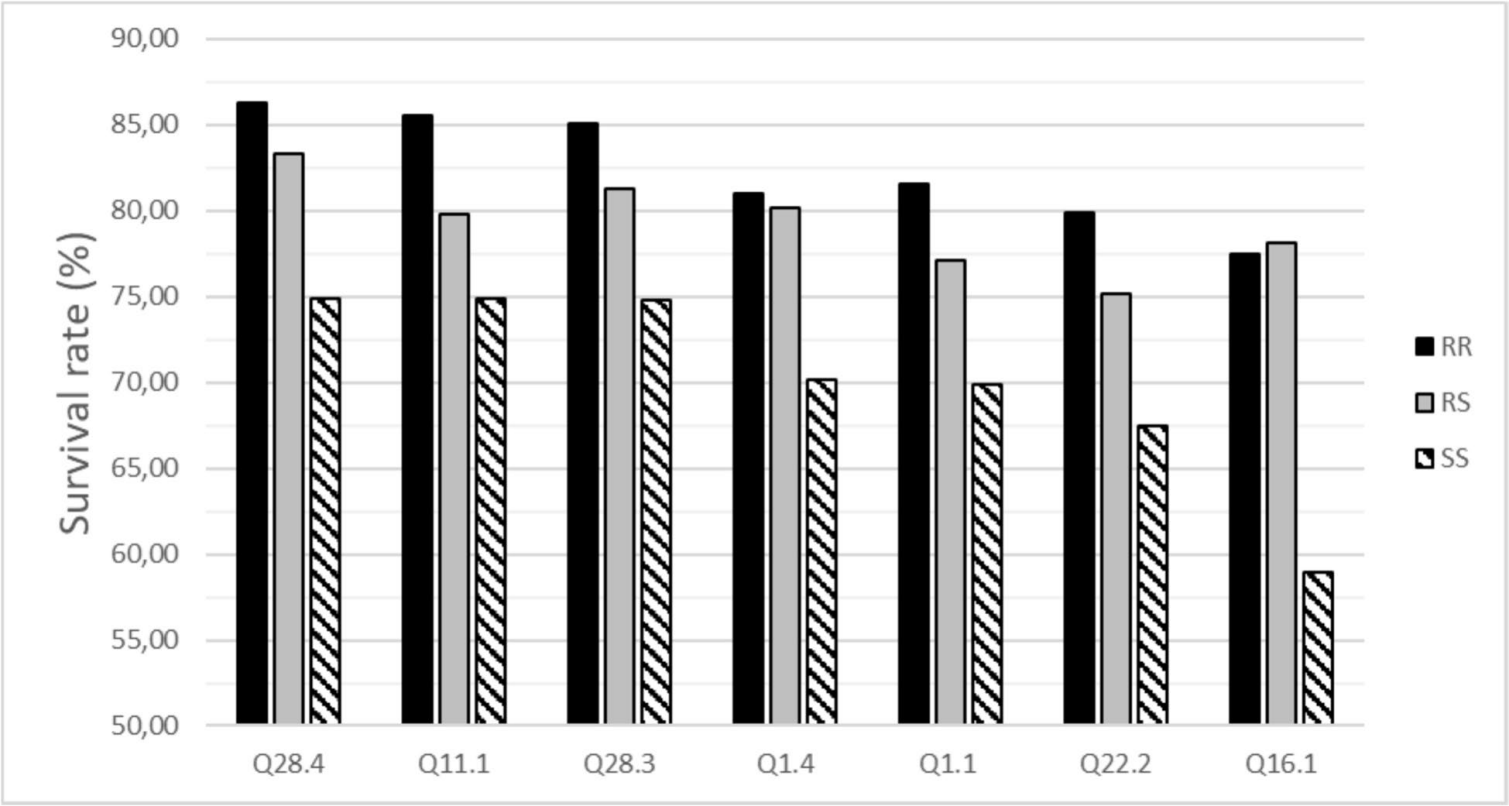
Average survival rate across the two generations for the double favorable homozygotes at the SNPs AX-578242608 (QTL Q1.6) and AX-564712030 (QTL Q16.3) according to their genotype at a third QTL

As the combination between Q1.6 and Q16.3 included very promising candidate genes and was also more present in the population than the very best combinations, we focused in Fig. 3 on the best 3-QTL favorable combinations for the double homozygous resistant genotype (RR-RR) on Q1.6 and Q16.3. We represented all 3 QTL-combinations that exhibited at least 10% higher mean survival rate for RR-RR-RR than for RR-RR-SS genotypes (Fig. 3). Regarding the mean survival rates of the triple RR combinations, the 3 top combinations (over 85% survival rate) involved Q28.4, Q11.1 and Q28.3, for which the minor alleles were beneficial (Table 3).

## Discussion

The heritability of IPN resistance in Norwegian and Chilean rainbow trout populations was previously estimated to be moderate [10, 12, 44], and our estimate was even a bit lower. Apart from random estimation errors, these small differences may be explained not only by genetic differences among the French population analyzed here and the two others, but also by important differences in the challenge protocols. First, the challenge was performed in separate tanks for each of the 200 families in the Norwegian study [12], while fish from all families were mixed and challenged together in ours and in the Chilean study. In addition, the average body weight at challenge was either lower (~ 0.2 g; [12]) or higher (~ 2.2 g; [10]) than in our study (~ 1.5 g). And last but not least, the challenge was by intraperitoneal inoculation in the Chilean case, while it was by bath in ours as well as in the Norwegian study. Recently a new estimate (h^2^ = 0.21), very close to ours, was reported in a limited full factorial mating design between 5 dams and 5 sires of the Osland Genetics strain, where 610 offspring were bath challenged [45]. Genetic variation among the viral strains used may also explain some differences in heritability of host disease resistance in the diverse challenges. While two different strains were used across years in our study, the heritability estimate was very similar between the two generations (results not shown). Therefore, we could expect limited noise linked to differences in strain virulence when comparing IPN resistance across the two generations of challenged fish.

Genomic selection is nowadays implemented in the main fish breeding programs to evaluate traits that cannot be measured directly on selection candidates, such as disease resistance traits. Its accuracy of selection is substantially higher than that of traditional pedigree selection based on sib-challenge tests [46, 47]. However, marker-assisted selection (MAS) can have major advantages (reduction of phenotyping and genotyping costs) over genomic selection for traits whose genetic variation is mainly explained by a few QTL. MAS can be applied to quickly eradicate breeders with detrimental genotypes from the selection nucleus, but it can also be used to only market eggs with a high genetic resistance score from multipliers. In our opinion, this last option is the one to favor to improve IPN resistance in rainbow trout production farms. Doing so, a breeding company will preserve long-term polygenic gains by maintaining more genetic diversity in the breeding population, while selling improved eggs from a limited set of breeders selected for their favorable genotypes on a few main QTL.

Our study confirmed the difference in the degree of polygenicity of resistance to IPNV between farmed populations of rainbow trout and Atlantic salmon. The major QTL for resistance to IPNV identified in the Atlantic salmon has not been found in rainbow trout. While a shared set of genes is likely involved in the biological pathways that underly host immune response to complex disease across populations or close species, their allele frequencies are expected to diverge due to natural selection associated with differences in pathogen exposure between populations and geographical areas [48]. It can be hypothesized that the 20 to 30 generations of natural selection of numerous farmed rainbow trout stocks since the first description of an IPN outbreak in 1940 [1] has contributed to generate the current genetic architecture with tens of loci with increased frequencies of beneficial alleles, in contrast to the segregation of a major gene, as observed in the limited number of Atlantic salmon strains that are farmed, which are only 10 to 15 generations removed from their wild ancestors since the start of breeding programs in the mid-1970s [49]. Resistance to IPNV in rainbow trout appears to be polygenic, although a limited number of SNPs (~ 63) explained three-quarters of the additive genetic variance in our French population. Those SNPs were associated with 25 QTL located on 10 of the 32 rainbow trout chromosomes. In particular, 5 QTL exhibited very significant effects, with differences in survival rates between homozygous genotypes ranging from over 20 to 30%. Three of these QTL were located on chromosome 1, while the other two were located on chromosome 16, *i.e.* positioned on the two chromosomes that were previously identified as carrying QTL for resistance to IPNV in rainbow trout [22, 26, 45, 50].

With regards to the previously detected QTL on chromosome 1, Santi et al. [26] indicated in their patent that homozygous fish for the cytosine allele at the most significant SNP AX-89929954 were expected to have a mean survival rate of 45% under conditions similar to those of the challenge test, while homozygous fish for the alternative allele and heterozygous fish had mean survival rates of 17 and 36%, respectively. The SNP AX-89929954 is located at 18.416812 Mb in the Arlee reference genome (USDA_OmykA_1.1.). The second and third most significant SNPs detected in [26] were AX-89918280 and AX-89938309, respectively located at 18.738017 Mb and 16.037298 Mb. All these three positions are located after the end of our last QTL region on chromosome 1 at 15.858 Mb, as well as far after the QTL identified in the Osland rainbow trout strain [50], with the top SNP AX-89932951 located at 12.200969 Mb on chromosome 1. Though this last SNP does not belong to the set of quality-filtered SNPs in our analysis, it is worth noting its location in-between our first two QTL on chromosome 1 (Q1.1 and Q1.2, Table 2). However, Ahmad et al. [45] did not confirm a significant association between the SNP AX-89932951 and IPN resistance in a validation cohort of the Osland strain. Differences in the QTL detected between studies may be due to limited statistical power, and to genetic differences across rainbow trout populations, but also to genetic differences in the virus strains. Tapia et al. [51] compared by RNA-seq the transcriptomic response of fry challenged with two Chilean isolates of IPNV, RTTX (genogroup 1), and ALKA (genogroup 5) in rainbow trout. The results revealed that infection with RTTX elicited an important modulation of the trout transcriptome compared to ALKA infection, with a greater number of highly differentially expressed genes compared to control fish, especially in the first week post-challenge. In the Norwegian studies on rainbow trout [45, 50] the virus strain (IPNV-R-L5 strain) used was isolated from Atlantic salmon.

In vitro, in a salmonid cell line, IPNV has been shown to block the type 1 interferon signaling pathway [52], resulting in limited production of the antiviral protein Mx protein, which is particularly effective against IPNV [53]. In Atlantic salmon, although clear induction of ISGs could be detected in experimental infections [54, 55], asymptomatic carrier states are usually observed in the field [56, 57]. The inhibition of both apoptosis and some innate antiviral components is a general hypothesis for IPNV-evading strategies, as reflected in vitro by the ability of IPNV to persist in cultured cell lines [58, 59] and in vivo by the establishment of an asymptomatic carrier stage. It is not clear how significant the carrier stage is for disease control strategies in rainbow trout farming [60, 61] but this may be important to evaluate, in particular in IPNV resistant selected fish.

Our GWAS pointed to a number of gene candidates on chromosomes 1, 2, 8, 11, 12, 13, 14, 16, 22, and 28. We identified some QTL on chromosome 8, 11, and 13, where some suggestive QTL were previously detected [45]. However, our top SNPs were at least 8 Mb removed from the top SNPs identified in [45]. In addition, the observed effects of our top SNPs between genotypes were limited (+ 7 to 13%, Table 3) for these QTL.

In our study, the most interesting QTL were associated with top SNPs with mean survival rate differences over 20% between the beneficial and detrimental genotypes. They were all associated with promising functional candidate genes, and were located on chromosome 1 (*uts2d* (ENSOMYG00000008336, LOC110534233) in Q1.1; *rc3h1* (ENSOMYG00000008605, LOC110488062) in Q1.6; *ga45b* (ENSOMYG00000000138, LOC100301708) in Q1.7), and on chromosome 16 (*irf2bpa* (ENSOMYG00000027318, LOC110491199) in Q16.2; *eif2ak2* (ENSOMYG00000027396, LOC100271898) in Q16.3).

*Urotensin-2* (*uts-2*) has pleiotropic effects on many physiological functions, including vasoconstriction, cell division, neuroendocrine activities, and inflammation in mammalian models. Pro-inflammatory actions of *uts-2* represent the most direct link with susceptibility to IPNV [41]. In mammals, UTS-2 stimulates a p38 MAPK dependent pro-inflammatory response mediated by TNFα, IL1β, IFNγ, and IL8 [62]. It also interacts with the IRF3/type I IFN pathway to modulate TNFα and IL1β levels in a p38 MAPK independent manner [63].

The E3 ubiquitin ligase ROQUIN-1 (*rc3h1* gene) has important anti-inflammatory functions and controls T cell responses through decay of targeted mRNAs. The ROQUIN protein binds to constitutive decay elements (CDE) or alternative decay elements (ADE) in the 3' UTR of mRNAs, leading to mRNA deadenylation and degradation. There are CDE in many key genes involved in T cell activation and inflammatory pathways, such as *hmgxb3, icos, ier3, nfkbid, nfkbiz, ppp1r10, tnf,* and *tnfrsf4/ox40* [64, 65]. Mutations in the mouse gene that encodes ROQUIN-1, which prevents interaction with its partner REGNASE-1, led to over-activation of T follicular helper cells, abnormal germinal center reaction, and auto-antibody production [41].

GADD45 (or GA45) proteins are stress sensors that respond to physiological and environmental stimulations, and could affect antiviral responses through modulation of inflammation or via more direct action on antiviral pathways. Indeed, GADD45B has an impact on chemotaxis of inflammatory cells, as reported for gadd45 ^−^/_-_ mice, which are unable to recruit granulocytes and macrophages in the peritoneal cavity after injection of bacterial lipopolysaccharides in this location [66]. Through interactions with the p38 MAPK and Janus kinase pathways, GADD45 proteins are also important for effectors and inflammatory functions of myeloid cells, such as oxidative burst, cytokine production, and phagocytosis [67]. Upon RNA virus infection, GADD45B protein interacts with Ras-GTPase-activating protein (SH3 domain)-binding protein 1 (G3BP1), contributing to stress granule formation and the stress granule-mediated type I IFN response, thereby directly linking these genes to the antiviral response [68]. It should be noted that all top SNPs in Q1.6 (associated with *rc3h1*) were in extremely high linkage disequilibrium with the single top SNP that defined Q1.7 and located in the *ga45b* gene (Supplementary see Additional file 1: Table S3), suggesting there may be cooperative actions of *rc3h1* and *ga45b* alleles.

The Q16.2 QTL was identified by a unique SNP located within the gene *mRNA-interferon regulatory factor 2-binding protein 2-A* (*irf2bp2a*) gene. In mammals, this transcriptional cofactor is involved in different biological systems and plays key roles in both lipid metabolism and control of inflammation [69]. IRF2BP2 specifically down-regulates innate inflammatory response of macrophages, and its expression is strongly repressed during differentiation of M1 inflammatory macrophages [70]. It is also involved in the regulation of lymphocyte activation [71]. Similar to genes associated with QTL on chromosome 1, the connexion of *irf2bp2a* to resistance and susceptibility to IPNV is likely through the modulation of inflammation and/or lymphocyte activation. However, there is no direct evidence about the associated mechanisms involved in immunity against (birna)viruses. This is in contrast with the gene *eif2ak2,* which is associated with Q16.3. The *eif2ak2* gene encodes a dsRNA-dependent serine/threonine-protein kinase (PKR) that phosphorylates the alpha subunit of eukaryotic translation initiation factor 2 (eIF2α). EIF2S1/eIF-2-alpha phosphorylation converts eIF2α into a global protein synthesis inhibitor, resulting in a shutdown of cellular and viral protein synthesis, while concomitantly initiating the preferential translation of ISR-specific mRNAs, such as the transcriptional activator ATF4 [72–74]. PKR expression is upregulated by viral infections and type I IFN [75], and plays a key role in the antiviral innate immune response in fish as well as in mammals (reviewed in [40]). A PKR role in rainbow trout response to IPNV infection has been reported, and in vitro loss of function demonstrated that salmonid PKR has conserved molecular functions in apoptosis and translation control [76]. Importantly, treatment with PKR inhibitors led to reduced IPNV titre in CHSE214 cells [77]. Moreover, IPNV infection does not upregulate PKR expression [78], most likely due to a global IPNV strategy of repression of type I IFN response to evade the host antiviral response. In fact, PKR can exert antiviral activity on a wide range of DNA and RNA viruses, including hepatitis C virus (HCV), hepatitis B virus (HBV), measles virus (MV), and herpes simplex virus 1 (HHV-1) [73, 79–84]. As an adapter protein and/or via its kinase activity, PKR triggers multiple signalling pathways, including p38 MAPK, NF-kappa-B, and insulin signalling pathways, controlling the expression of pro-inflammatory cytokines and IFNs [85–87].

Apart from [44], where AX-89961019 was detected as a suggestive QTL (located at 6.932350 Mb on Arlee reference chromosome 16), only the very first QTL studies on a limited number of Japanese families of rainbow trout detected a QTL on chr16 [21, 22], but the few microsatellites used at that time do not allow us to confirm whether the same genomic region is detected in our study. Our two main QTL on chromosome 16 (Q16.2 and Q16.3) are located in a run of homozygosity (ROH) island of 2174 kb that was previously identified in the LB population [88], between 1.504245 and 3.678601 Mb. This LB population corresponds to an earlier generation of selection of the same commercial population used in our current study. However, this ROH region was not shared with the 3 other populations considered in [88]. ROH island identifies a region of the genome that is frequently homozygous, suggesting a signature of positive selection [89]. This was confirmed in the current study, as the 3 top SNPs located in Q16.2 and Q16.3 were included in a ROH for 37 to 45% of the fish used (results not shown). The question may arise whether Q16.2 and Q16.3 correspond to a cluster of two genes working together as a supergene [90] to provide an integrated control of a complex response to IPNV.

## Conclusions

Based on functional information from the literature, various promising candidate genes for resistance to IPNV were identified, orthologous to genes that control type I IFN response or inflammation response, as well as involved in diverse roles in the cells which might be connected to the virus cycle or to antiviral processes. Remarkably, genes associated with the most significant QTL on chromosomes 1 and 16 are all involved in regulation of inflammatory pathways, such as the p38 MAPK pathway, strongly suggesting a central role of inflammation in resistance and susceptibility to IPNV in rainbow trout, with PKR being a key factor.

These QTL offer the possibility of marker-assisted selection of breeders used by multipliers for rapid dissemination of genetic improvement for IPN resistance in rainbow trout, similar to what has occurred for the last 15 years in Atlantic salmon, significantly reducing mortality in salmonid aquaculture.

## Supplementary Information

Additional file 1.

## Data Availability

The datasets generated during and/or analysed during the current study are not publicly available because they parly belong to a commercial breeding company, but are available from the corresponding author on reasonable request and with permission of the relevant company.

## References

[1] M’Gonigle RH. Acute catarrhal enteritis of salmonid fingerlings. Trans Am Fish Soc. 1941;70:297–303.

[2] Besse P, Kinkelin PD, Donon O. Sur l’existence en France de la nécrose pancréatique de la truite arc-en-ciel (*Salmo gairdneri*). (Note préliminaire). Bull Acad Vet Fr. 1965;118:185–92. 5837804

[3] Roberts RJ, Pearson MD. Infectious pancreatic necrosis in Atlantic salmon, *Salmo salar* L. J Fish Dis. 2005;28:383–90. 16083443 10.1111/j.1365-2761.2005.00642.x

[4] Tapia D, Kuznar J, Farlora R, Yáñez JM. Differential transcriptomic response of rainbow trout to infection with two strains of IPNV. Viruses. 2021;14:21. 35062225 10.3390/v14010021 PMC8780770

[5] Dopazo CP. The infectious pancreatic necrosis virus (IPNV) and its virulence determinants: what is known and what should be known. Pathogens. 2020;9:94. 32033004 10.3390/pathogens9020094 PMC7168660

[6] Duan K, Tang X, Zhao J, Ren G, Shao Y, Lu T, et al. An inactivated vaccine against infectious pancreatic necrosis virus in rainbow trout (*Oncorhynchus mykiss*). Fish Shellfish Immunol. 2022;127:48–55. 35697270 10.1016/j.fsi.2022.06.008

[7] Li L, Liu W, Zhang Z, Zhao J, Lu T, Shao Y, et al. IPNV inactive vaccine supplemented with GEL 02 PR adjuvant: protective efficacy, cross-protection, and stability. Fish Shellfish Immunol. 2025;158:110167. 39890040 10.1016/j.fsi.2025.110167

[8] Li S, Li X, Yuan R, Chen X, Chen S, Qiu Y, et al. Development of a recombinant adenovirus-vectored vaccine against both infectious hematopoietic necrosis virus and infectious pancreatic necrosis virus in rainbow trout (*Oncorhynchus mykiss*). Fish Shellfish Immunol. 2023;132:108457. 36455780 10.1016/j.fsi.2022.108457

[9] Yáñez JM, Houston RD, Newman S. Genetics and genomics of disease resistance in salmonid species. Front Genet. 2014;5:415. 25505486 10.3389/fgene.2014.00415 PMC4245001

[10] Flores-Mara R, Rodríguez FH, Bangera R, Lhorente JP, Neira R, Newman S, et al. Resistance against infectious pancreatic necrosis exhibits significant genetic variation and is not genetically correlated with harvest weight in rainbow trout (*Oncorhynchus mykiss*). Aquaculture. 2017;479:155–60.

[11] Rodríguez FH, Flores-Mara R, Yoshida GM, Barría A, Jedlicki AM, Lhorente JP, et al. Genome-wide association analysis for resistance to infectious pancreatic necrosis virus identifies candidate genes involved in viral replication and immune response in rainbow trout (*Oncorhynchus mykiss*). G3 Bethesda. 2019;9:2897–904. 31324747 10.1534/g3.119.400463 PMC6723134

[12] Wetten M, Kjøglum S, Fjalestad KT, Skjaervik O, Storset A. Genetic variation in resistance to infectious pancreatic necrosis in rainbow trout (*Oncorhynchus mykiss*) after a challenge test: breeding for IPN-resistance in rainbow trout. Aquac Res. 2011;42:1745–51.

[13] Houston RD, Haley CS, Hamilton A, Guy DR, Tinch AE, Taggart JB, et al. Major quantitative trait loci affect resistance to infectious pancreatic necrosis in Atlantic salmon (*Salmo salar*). Genetics. 2008;178:1109–15. 18245341 10.1534/genetics.107.082974 PMC2248365

[14] Moen T, Baranski M, Sonesson AK, Kjøglum S. Confirmation and fine-mapping of a major QTL for resistance to infectious pancreatic necrosis in Atlantic salmon (*Salmo salar*): population-level associations between markers and trait. BMC Genomics. 2009;10:368. 19664221 10.1186/1471-2164-10-368 PMC2728743

[15] Houston RD, Haley CS, Hamilton A, Guy DR, Mota-Velasco JC, Gheyas AA, et al. The susceptibility of Atlantic salmon fry to freshwater infectious pancreatic necrosis is largely explained by a major QTL. Heredity. 2010;105:318–27. 19935825 10.1038/hdy.2009.171

[16] Moen T, Torgersen J, Santi N, Davidson WS, Baranski M, Ødegård J, et al. Epithelial cadherin determines resistance to infectious pancreatic necrosis virus in Atlantic salmon. Genetics. 2015;200:1313–26. 26041276 10.1534/genetics.115.175406 PMC4574245

[17] Bishop SC, Woolliams JA. Genomics and disease resistance studies in livestock. Livest Sci. 2014;166:190–8. 26339300 10.1016/j.livsci.2014.04.034 PMC4547482

[18] Moen T, Ødegård J. Genomics in selective breeding of Atlantic salmon. In: Proceedings of the 10th World Congress on Genetics Applied to Livestock Production: 17–22 August 2014; Vancouver. 2014.

[19] Pavelin J, Jin YH, Gratacap RL, Taggart JB, Hamilton A, Verner-Jeffreys DW, et al. The nedd-8 activating enzyme gene underlies genetic resistance to infectious pancreatic necrosis virus in Atlantic salmon. Genomics. 2021;113:3842–50. 34547402 10.1016/j.ygeno.2021.09.012 PMC8682971

[20] Hillestad B, Johannessen S, Melingen GO, Moghadam HK. Identification of a new infectious pancreatic necrosis virus (IPNV) variant in Atlantic salmon (*Salmo salar L.*) that can cause high mortality even in genetically resistant fish. Front Genet. 2021;12:635185. 34899819 10.3389/fgene.2021.635185 PMC8663487

[21] Ozaki A, Khoo S-K, Yoshiura Y, Ototake M, Sakamoto T, Dijkstra JM, et al. Identification of additional quantitative trait loci (QTL) responsible for susceptibility to infectious pancreatic necrosis virus in rainbow trout. Fish Pathol. 2007;42:131–40.

[22] Ozaki A, Sakamoto T, Khoo S, Nakamura K, Coimbra MR, Akutsu T, et al. Quantitative trait loci (QTLs) associated with resistance/susceptibility to infectious pancreatic necrosis virus (IPNV) in rainbow trout (*Oncorhynchus mykiss*). Mol Gen Genomics. 2001;265:23–31. 10.1007/s004380000392 11370869

[23] Phillips RB, Nichols KM, DeKoning JJ, Morasch MR, Keatley KA, Rexroad C, et al. Assignment of rainbow trout linkage groups to specific chromosomes. Genetics. 2006;174:1661–70. 16951085 10.1534/genetics.105.055269 PMC1667062

[24] Danzmann RG, Cairney M, Davidson WS, Ferguson MM, Gharbi K, Guyomard R, et al. A comparative analysis of the rainbow trout genome with 2 other species of fish (Arctic charr and Atlantic salmon) within the tetraploid derivative Salmonidae family (subfamily: Salmoninae). Genome. 2005;48:1037–51. 16391673 10.1139/g05-067

[25] Pearse DE, Barson NJ, Nome T, Gao G, Campbell MA, Abadía-Cardoso A, et al. Sex-dependent dominance maintains migration supergene in rainbow trout. Nat Ecol Evol. 2019;3:1731–42. 31768021 10.1038/s41559-019-1044-6

[26] Santi N, Moen T, Ødegård J. Method for predicting resistance. Patent application publication US2019/0241980A1; 2019.

[27] Gao G, Magadan S, Waldbieser GC, Youngblood RC, Wheeler PA, Scheffler BE, et al. A long reads-based *de-novo* assembly of the genome of the Arlee homozygous line reveals chromosomal rearrangements in rainbow trout. G3 (Bethesda). 2021;11:052. 10.1093/g3journal/jkab052 PMC8763230 33616628

[28] Bernard M, Dehaullon A, Gao G, Paul K, Lagarde H, Charles M, et al. Development of a high-density 665 K SNP array for rainbow trout genome-wide genotyping. Front Genet. 2022;13:941340. 35923696 10.3389/fgene.2022.941340 PMC9340366

[29] Chang CC, Chow CC, Tellier LC, Vattikuti S, Purcell SM, Lee JJ. Second-generation PLINK: rising to the challenge of larger and richer datasets. Gigascience. 2015;4:s13742–015-0047–8. 10.1186/s13742-015-0047-8 PMC4342193 25722852

[30] Purcell S, Neale B, Todd-Brown K, Thomas L, Ferreira MAR, Bender D, et al. PLINK: a tool set for whole-genome association and population-based linkage analyses. Am J Hum Genet. 2007;81:559–75. 17701901 10.1086/519795 PMC1950838

[31] Griot R, Allal F, Brard-Fudulea S, Morvezen R, Haffray P, Phocas F, et al. APIS: an auto-adaptive parentage inference software that tolerates missing parents. Mol Ecol Resour. 2020;20:579–90. 31609085 10.1111/1755-0998.13103

[32] Roche J, Griot R, Allal F, Besson M, Haffray P, Patrice P, et al. APIS: an updated parentage assignment software managing triploids induced from diploid parents. G3: Genes, Genomes, Genetics. 2024;14:jkae143. 38954534 10.1093/g3journal/jkae143 PMC11304945

[33] Sargolzaei M, Chesnais JP, Schenkel FS. A new approach for efficient genotype imputation using information from relatives. BMC Genomics. 2014;15:478. 24935670 10.1186/1471-2164-15-478 PMC4076979

[34] Misztal I, Tsuruta S, Lourenco D, Masuda Y, Aguilar I, Legarra A, et al. Manual for BLUPF90 family of programs. 2014. http://nce.ads.uga.edu/wiki/lib/exe/fetch.php?media=blupf90_all7.pdf. Accessed 26 Feb 2025.

[35] Van Raden PM. Efficient methods to compute genomic predictions. J Dairy Sci. 2008;91:4414–23. 18946147 10.3168/jds.2007-0980

[36] Zhou X, Carbonetto P, Stephens M. Polygenic modeling with Bayesian sparse linear mixed models. PLoS Genet. 2013;9:e1003264. 23408905 10.1371/journal.pgen.1003264 PMC3567190

[37] Stephens M, Balding DJ. Bayesian statistical methods for genetic association studies. Nat Rev Genet. 2009;10:681–90. 19763151 10.1038/nrg2615

[38] Kass RE, Raftery AE. Bayes factors. J Am Stat Assoc. 1995;90:773–95.

[39] Michenet A, Saintilan R, Venot E, Phocas F. Insights into the genetic variation of maternal behavior and suckling performance of continental beef cows. Genet Sel Evol. 2016;48:45. 27335091 10.1186/s12711-016-0223-z PMC4918023

[40] Chaumont L, Collet B, Boudinot P. Double-stranded RNA-dependent protein kinase (PKR) in antiviral defence in fish and mammals. Dev Comp Immunol. 2023;145:104732. 37172664 10.1016/j.dci.2023.104732

[41] Sun S, Liu L. Urotensin II: an inflammatory cytokine. J Endocrinol. 2019;240:R107–17. 30601760 10.1530/JOE-18-0505

[42] Behrens G, Edelmann SL, Raj T, Kronbeck N, Monecke T, Davydova E, et al. Disrupting Roquin-1 interaction with Regnase-1 induces autoimmunity and enhances antitumor responses. Nat Immunol. 2021;22:1563–76. 34811541 10.1038/s41590-021-01064-3 PMC8996344

[43] Essig K, Kronbeck N, Guimaraes JC, Lohs C, Schlundt A, Hoffmann A, et al. Roquin targets mRNAs in a 3′-UTR-specific manner by different modes of regulation. Nat Commun. 2018;9:3810. 30232334 10.1038/s41467-018-06184-3 PMC6145892

[44] Yoshida GM, Carvalheiro R, Rodríguez FH, Lhorente JP, Yáñez JM. Single-step genomic evaluation improves accuracy of breeding value predictions for resistance to infectious pancreatic necrosis virus in rainbow trout. Genomics. 2019;111:127–32. 29357303 10.1016/j.ygeno.2018.01.008

[45] Ahmad A, Aslam ML, Evensen Ø, Gamil AAA, Berge A, Solberg T, et al. The genetics of resistance to infectious pancreatic necrosis virus in rainbow trout unveiled through survival and virus load data. Front Genet. 2024;15:1484287. 39628812 10.3389/fgene.2024.1484287 PMC11611855

[46] Sonesson AK, Meuwissen TH. Testing strategies for genomic selection in aquaculture breeding programs. Genet Sel Evol. 2009;41:37. 19566932 10.1186/1297-9686-41-37 PMC2714299

[47] Villanueva B, Fernández J, García-Cortés LA, Varona L, Daetwyler HD, Toro MA. Accuracy of genome-wide evaluation for disease resistance in aquaculture breeding programs1. J Anim Sci. 2011;89:3433–42. 21742941 10.2527/jas.2010-3814

[48] Randolph HE, Aracena KA, Lin Y, Mu Z, Barreiro LB. Shaping immunity: The influence of natural selection on population immune diversity. Immunol Rev. 2024;323:227–40. 38577999 10.1111/imr.13329

[49] Gjedrem T. Genetic improvement for the development of efficient global aquaculture: a personal opinion review. Aquaculture. 2012;344–349:12–22.

[50] Aslam M, Valdemarsson S, Berg A, Gjerde B. GWAS and accuracy of predictions for resistance against IPN in rainbow trout. In: Book of abstracts of the 70th annual meeting of the European federation of animal science: 26–30 August 2019; Ghent. 2019.

[51] Tapia D, Eissler Y, Reyes-Lopez FE, Kuznar J, Yáñez JM. Infectious pancreatic necrosis virus in salmonids: molecular epidemiology and host response to infection. Rev Aquacult. 2022;14:751–69.

[52] Collet B. Innate immune responses of salmonid fish to viral infections. Dev Comp Immunol. 2014;43:160–73. 23981327 10.1016/j.dci.2013.08.017

[53] Lester K, Hall M, Urquhart K, Gahlawat S, Collet B. Development of an in vitro system to measure the sensitivity to the antiviral Mx protein of fish viruses. J Virol Methods. 2012;182:1–8. 22405879 10.1016/j.jviromet.2012.01.014

[54] Ellis AE, Cavaco A, Petrie A, Lockhart K, Snow M, Collet B. Histology, immunocytochemistry and qRT-PCR analysis of Atlantic salmon, *Salmo salar L.*, post-smolts following infection with infectious pancreatic necrosis virus (IPNV). J Fish Dis. 2010;33:803–18. 20561142 10.1111/j.1365-2761.2010.01174.x

[55] Mcbeath A, Snow M, Secombes C, Ellis A, Collet B. Expression kinetics of interferon and interferon-induced genes in Atlantic salmon (*Salmo salar*) following infection with infectious pancreatic necrosis virus and infectious salmon anaemia virus. Fish Shellfish Immunol. 2007;22:230–41. 16806972 10.1016/j.fsi.2006.05.004

[56] Lockhart K, Gahlawat SK, Soto-Mosquera D, Bowden TJ, Ellis AE. IPNV carrier Atlantic salmon growers do not express Mx mRNA and poly I:C-induced Mx response does not cure the carrier state. Fish Shellfish Immunol. 2004;17:347–52. 15312661 10.1016/j.fsi.2004.04.011

[57] Ørpetveit I, Mikalsen AB, Sindre H, Evensen Ø, Dannevig BH, Midtlyng PJ. Detection of infectious pancreatic necrosisvirus in subclinically infected Atlantic salmon by virus isolation in cell culture or real-time reverse transcription polymerase chain reaction: influence of sample preservation and storage. J Vet Diagn Invest. 2010;22:886–95. 21088171 10.1177/104063871002200606

[58] Jurado MT, García-Valtanen P, Estepa A, Perez L. Antiviral activity produced by an IPNV-carrier EPC cell culture confers resistance to VHSV infection. Vet Microbiol. 2013;166:412–8. 23891172 10.1016/j.vetmic.2013.06.022

[59] Rodríguez Saint-Jean S, De Las Heras AI, Pérez Prieto SI. The persistence of infectious pancreatic necrosis virus and its influence on the early immune response. Vet Immunol Immunopathol. 2010;136:81–91. 20334936 10.1016/j.vetimm.2010.02.015

[60] Ahne W, Thomsen I. Infectious pancreatic necrosis: detection of virus and antibodies in rainbow trout IPNV-carrier (*Salmo gairdneri*). Zentralbl Veterinarmed B. 1986;33:552–4. 3811691 10.1111/j.1439-0450.1986.tb00067.x

[61] Rodriguez S, Alonso M, Perez-Prieto SI. Detection of infections pancreatic necrosis virus (IPNV) from leukocytes of carrier rainbow trout *Oncorhynchus mykiss*. Fish Pathol. 2001;36:139–46.

[62] Liu LM, Liang DY, Ye CG, Tu WJ, Zhu T. The UII/UT system mediates upregulation of proinflammatory cytokines through p38 MAPK and NF-κB pathways in LPS-stimulated Kupffer cells. PLoS ONE. 2015;10:e0121383. 25803040 10.1371/journal.pone.0121383 PMC4372515

[63] Liu L, Tu W, Zhu T, Wang X, Tan Z, Zhong H, et al. IRF3 is an important molecule in the UII/UT system and mediates immune inflammatory injury in acute liver failure. Oncotarget. 2016;7:49027–41. 27448985 10.18632/oncotarget.10717 PMC5226488

[64] Tan D, Zhou M, Kiledjian M, Tong L. The ROQ domain of Roquin recognizes mRNA constitutive-decay element and double-stranded RNA. Nat Struct Mol Biol. 2014;21:679–85. 25026078 10.1038/nsmb.2857 PMC4125485

[65] Tavernier SJ, Athanasopoulos V, Verloo P, Behrens G, Staal J, Bogaert DJ, et al. Author correction: a human immune dysregulation syndrome characterized by severe hyperinflammation with a homozygous nonsense Roquin-1 mutation. Nat Commun. 2019;10:5337. 31745085 10.1038/s41467-019-13379-9 PMC6864049

[66] Salerno DM, Tront JS, Hoffman B, Liebermann DA. Gadd45a and Gadd45b modulate innate immune functions of granulocytes and macrophages by differential regulation of p38 and JNK signaling. J Cell Physiol. 2012;227:3613–20. 22307729 10.1002/jcp.24067

[67] Hoffman B, Liebermann DA. Gadd45 modulation of intrinsic and extrinsic stress responses in myeloid cells. J Cell Physiol. 2009;218:26–31. 18780287 10.1002/jcp.21582

[68] Chathuranga WAG, Nikapitiya C, Kim J-H, Chathuranga K, Weerawardhana A, Dodantenna N, et al. Gadd45β is critical for regulation of type I interferon signaling by facilitating G3BP-mediated stress granule formation. Cell Rep. 2023;42:113358. 37917584 10.1016/j.celrep.2023.113358

[69] Chen H-H, Keyhanian K, Zhou X, Vilmundarson RO, Almontashiri NAM, Cruz SA, et al. Irf2bp2 reduces macrophage inflammation and susceptibility to atherosclerosis. Circ Res. 2015;117:671–83. 26195219 10.1161/CIRCRESAHA.114.305777

[70] Zhang H, Reilly MP. IRF2BP2: a new pPlayer at the crossroads of inflammation and lipid metabolism. Circ Res. 2015;117:656–8. 26405180 10.1161/CIRCRESAHA.115.307245 PMC4862198

[71] Ramalho-Oliveira R, Oliveira-Vieira B, Viola JPB. IRF2BP2: a new player in the regulation of cell homeostasis. J Leukoc Biol. 2019;106:717–23. 31022319 10.1002/JLB.MR1218-507R

[72] Harashima A, Guettouche T, Barber GN. Phosphorylation of the NFAR proteins by the dsRNA-dependent protein kinase PKR constitutes a novel mechanism of translational regulation and cellular defense. Genes Dev. 2010;24:2640–53. 21123651 10.1101/gad.1965010 PMC2994038

[73] Kang J-I, Kwon S-N, Park S-H, Kim YK, Choi S-Y, Kim JP, et al. PKR protein kinase is activated by hepatitis C virus and inhibits viral replication through translational control. Virus Res. 2009;142:51–6. 19189853 10.1016/j.virusres.2009.01.007

[74] Okumura F, Okumura AJ, Uematsu K, Hatakeyama S, Zhang D-E, Kamura T. Activation of double-stranded RNA-activated protein kinase (PKR) by interferon-stimulated gene 15 (ISG15) modification down-regulates protein translation. J Biol Chem. 2013;288:2839–47. 23229543 10.1074/jbc.M112.401851 PMC3554948

[75] Meurs E, Chong K, Galabru J, Thomas NSB, Kerr IM, Williams BRG, et al. Molecular cloning and characterization of the human double-stranded RNA-activated protein kinase induced by interferon. Cell. 1990;62:379–90. 1695551 10.1016/0092-8674(90)90374-n

[76] Chaumont L, Peruzzi M, Huetz F, Raffy C, Le Hir J, Minke J, et al. Salmonid double-stranded RNA–dependent protein kinase activates apoptosis and inhibits protein synthesis. J Immunol. 2024;213:700–17. 39058317 10.4049/jimmunol.2400076

[77] Gamil A, Xu C, Mutoloki S, Evensen Ø. PKR activation favors infectious pancreatic necrosis virus replication in infected cells. Viruses. 2016;8:173. 27338445 10.3390/v8060173 PMC4926193

[78] Gamil A, Mutoloki S, Evensen Ø. A piscine birnavirus induces inhibition of protein synthesis in CHSE-214 cells primarily through the induction of eIF2α phosphorylation. Viruses. 2015;7:1987–2005. 25885006 10.3390/v7041987 PMC4411686

[79] Cassady KA, Gross M. The *Herpes simplex* virus type 1 U_S_ 11 protein interacts with protein kinase R in infected cells and requires a 30-amino-acid sequence adjacent to a kinase substrate domain. J Virol. 2002;76:2029–35. 11836380 10.1128/jvi.76.5.2029-2035.2002 PMC135940

[80] Chang J-H, Kato N, Muroyama R, Taniguchi H, Guleng B, Dharel N, et al. Double-stranded RNA-activated protein kinase inhibits hepatitis C virus replication but may be not essential in interferon treatment. Liver Int. 2010;30:311–8. 19840259 10.1111/j.1478-3231.2009.02144.x

[81] Lin SS, Lee DCW, Law AHY, Fang JW, Chua DTT, Lau ASY. A role for protein kinase PKR in the mediation of Epstein-Barr virus latent membrane protein-1-induced IL-6 and IL-10 expression. Cytokine. 2010;50:210–9. 20171114 10.1016/j.cyto.2010.01.008

[82] Okonski KM, Samuel CE. Stress granule formation induced by Measles virus is protein kinase PKR dependent and impaired by RNA adenosine deaminase ADAR1. J Virol. 2013;87:756–66. 23115276 10.1128/JVI.02270-12 PMC3554044

[83] Park I-H, Baek K-W, Cho E-Y, Ahn B-Y. PKR-dependent mechanisms of interferon-α for inhibiting hepatitis B virus replication. Mol Cells. 2011;32:167–72. 21710204 10.1007/s10059-011-1059-6 PMC3887671

[84] Zhang L, Alter HJ, Wang H, Jia S, Wang E, Marincola FM, et al. The modulation of hepatitis C virus 1a replication by PKR is dependent on NF-kB mediated interferon beta response in Huh7.5.1 cells. Virology. 2013;438:28–36. 23399035 10.1016/j.virol.2013.01.015 PMC3594529

[85] Li Y, Xie J, Wu S, Xia J, Zhang P, Liu C, et al. Protein kinase regulated by dsRNA downregulates the interferon production in dengue virus- and dsRNA-stimulated human lung epithelial cells. PLoS ONE. 2013;8:e55108. 23372823 10.1371/journal.pone.0055108 PMC3555826

[86] McAllister CS, Taghavi N, Samuel CE. Protein kinase PKR amplification of interferon β induction occurs through initiation factor eIF-2α-mediated translational control. J Biol Chem. 2012;287:36384–92. 22948139 10.1074/jbc.M112.390039 PMC3476304

[87] Shen S, Niso-Santano M, Adjemian S, Takehara T, Malik SA, Minoux H, et al. Cytoplasmic STAT3 represses autophagy by inhibiting PKR activity. Mol Cell. 2012;48:667–80. 23084476 10.1016/j.molcel.2012.09.013

[88] Paul K, Restoux G, Phocas F. Genome-wide detection of positive and balancing signatures of selection shared by four domesticated rainbow trout populations (*Oncorhynchus mykiss*). Genet Sel Evol. 2024;56:13. 38389056 10.1186/s12711-024-00884-9 PMC10882880

[89] Saravanan KA, Panigrahi M, Kumar H, Parida S, Bhushan B, Gaur GK, et al. Genomic scans for selection signatures revealed candidate genes for adaptation and production traits in a variety of cattle breeds. Genomics. 2021;113:955–63. 33610795 10.1016/j.ygeno.2021.02.009

[90] Schwander T, Libbrecht R, Keller L. Supergenes and complex phenotypes. Curr Biol. 2014;24:R288–94. 24698381 10.1016/j.cub.2014.01.056

